# 4mCPred-MTL: Accurate Identification of DNA 4mC Sites in Multiple Species Using Multi-Task Deep Learning Based on Multi-Head Attention Mechanism

**DOI:** 10.3389/fcell.2021.664669

**Published:** 2021-05-10

**Authors:** Rao Zeng, Song Cheng, Minghong Liao

**Affiliations:** ^1^Department of Software Engineering, School of Informatics, Xiamen University, Xiamen, China; ^2^Department of Thoracic Surgery, Heilongjiang Province Land Reclamation Headquarters General Hospital, Harbin, China

**Keywords:** multi-task learning, feature sharing, DNA 4mC modification, epigenetics, deep learning, transformer

## Abstract

DNA methylation is one of the most extensive epigenetic modifications. DNA 4mC modification plays a key role in regulating chromatin structure and gene expression. In this study, we proposed a generic 4mC computational predictor, namely, 4mCPred-MTL using multi-task learning coupled with Transformer to predict 4mC sites in multiple species. In this predictor, we utilize a multi-task learning framework, in which each task is to train species-specific data based on Transformer. Extensive experimental results show that our multi-task predictive model can significantly improve the performance of the model based on single task and outperform existing methods on benchmarking comparison. Moreover, we found that our model can sufficiently capture better characteristics of 4mC sites as compared to existing commonly used feature descriptors, demonstrating the strong feature learning ability of our model. Therefore, based on the above results, it can be expected that our 4mCPred-MTL can be a useful tool for research communities of interest.

## Introduction

Epigenetics refers to the reversible and heritable changes in gene function when there is no change in the nuclear DNA sequence (Zuo et al., 2020). Epigenetic phenomena include DNA methylation, RNA interference, histone modification, etc. (Tang W. et al., 2018; Wang et al., 2018; Liu et al., 2019; Hong et al., 2020; Lv et al., 2020a; Zhang D. et al., 2020; Min et al., 2021). Among them, DNA methylation is one of the most extensive epigenetic modifications (Zhu et al., 2019). It is a form of DNA chemical modification that can change genetic performance without changing the DNA sequence. DNA methylation refers to the binding of a methyl group to the cytosine 5 carbon covalent bond of genomic CpG dinucleotides under the action of DNA methyltransferase (Jin et al., 2011; Lv et al., 2020b). A large number of studies have shown that DNA methylation can cause changes in chromatin structure, DNA conformation, DNA stability, and the way that DNA interacts with proteins, thereby controlling gene expression (Jin et al., 2011; Zeng et al., 2016; Zhang et al., 2019; Luo et al., 2020; Shen and Zou, 2020). DNA 4mC has been reported as an effective DNA modification, which can protect its own DNA from restriction enzyme-mediated degradation (Chen et al., 2017; Wei et al., 2019b). Currently, we have relatively little knowledge regarding 4mC modifications. In order to further study its regulatory mechanism and its biological impact on the organism, it is critical to identify the distribution of 4mC sites in the whole genome.

With the development of high-throughput sequencing technology, 4mC sites can be effectively identified through web-lab biochemical experiments (Flusberg et al., 2010), but this kind of method is time-consuming and labor-intensive. Therefore, it is necessary to develop a computational model that can efficiently and accurately predict and identify 4mC sites. Chen et al. (2017) first developed a tool, namely, iDNA4mC for predicting 4mC sites by establishing a feature set based on chemical properties and occurrence frequency of nucleotides and training a support vector machine (SVM)-based predicting model. In order to take into account more of the physical and chemical properties of DNA, He et al. (2018) proposed 4mCPred, also an SVM-based predictor that used position-specific trinucleotide propensity (PSTNP) and electron–ion interaction potential (EIIP) for feature extraction. In particular, they further optimize the features based on F-score to enhance the generalization ability of the model. Similarly, through four feature coding schemes and using two-step feature optimization method, Wei et al. (2019a) constructed a prediction model called 4mCPred-SVM, which is shown to perform better than previous methods on benchmarking comparison. Later, Manavalan et al. (2019b) first proposed the meta-predictor Meta-4mCpred for predicting 4mC sites. It used a variety of feature extraction methods to convert DNA sequences into a total of 14 feature descriptors and trained four different classifiers. Particularly, meta-4mCpred exhibits good performance with independent test, demonstrating the excellent generalization ability. To make full use of the advantages of each prediction method mentioned above, Tang et al. (2020) developed DNA4mC-LIP, which for the first time linearly integrated all the previous methods for the 4mC site prediction. In recent years, deep learning has been widely used in the field of bioinformatics. Xu et al. (2020) developed the first deep learning Deep4mC, which converted sequences into digital vectors through binary, enhanced nucleic acid composition (ENAC), EIIP, and nucleotide chemical property (NCP) feature encoding schemes and inputted them into two convolutional layers without pooling layers and the attention layers. The average area under the ROC (receiver operating characteristic) curve (AUC) values of its prediction for multiple species were greater than 0.9 in multiple cross-validations. In our previous work, we proposed a two-layer deep learning model called Deep4mcPred, which utilizes a hybrid network of ResNet and long short-term memory (LSTM) (Zeng and Liao, 2020).

Although much progress has been made by the methods mentioned above, the performance is still not satisfactory. Moreover, most existing predictors are designed for one specific species. Although they provide a cross-species model and validation test, the performance is always not that good as compared to the original species-specific model. Therefore, to address this problem, we established a generic 4mC predictor, namely, 4mCPred-MTL using multi-task learning coupled with Transformer, which is a widely used NLP (natural language processing) technique, to predict 4mC sites in multiple species. In this predictor, we utilize a multi-task learning framework, in which each task is to train species-specific data based on Transformer. Extensive experimental results show that our multi-task predictive model can significantly improve the performance of the model based on a single task and outperform existing methods. Moreover, we found that the feature representations learned from our model can capture better characteristics of 4mC sites as compared to the existing commonly used feature descriptors, demonstrating the strong feature learning ability. Therefore, based on the above results, it can be expected that our 4mCPred-MTL can be a useful tool for research communities of interest.

## Materials and Methods

### Datasets

Previous studies have demonstrated that a stringent dataset is essential for building a robust predictive model (Liang et al., 2017; Zeng and Liao, 2020; Su et al., 2021). In our previous work (Zeng and Liao, 2020), we constructed large-scale datasets for three species, including *Arabidopsis thaliana* (*A. thaliana*), *Caenorhabditis elegans* (*C. elegans*), and *Drosophila melanogaster* (*D. melanogaster*). As for the positive samples, there are 20,000 positive samples, and each sample is a 41-bp-long sequence centered with true 4mC sites. Similarly, the dataset contains the same number of negative samples, which are cytosine-centered sequences with lengths of 41 bp but are not recognized by the single-molecule, real-time (SMRT) sequencing technology.

#### Training Set and Independent Test Set

Considering the performance, most of the existing predictors are evaluated by cross validation test, which might produce performance bias; we here randomly split the datasets into (Zuo et al., 2020) training set for model training and evaluation and (Liu et al., 2019) independent test set for model robustness validation. Thus, we randomly divided the dataset into training set and testing set with the ratio of 8:2, resulting in 16,000 samples in the training set and 4,000 samples in the testing set. The details of the datasets are presented in Table 1. Notably, for fair comparison, all the existing methods are evaluated on the test set.

**TABLE 1:** Summary of benchmark datasets in three species.

Species	Training set		Testing set	
		
	Positives	Negatives	Positives	Negatives
*A. thaliana*	16,000	16,000	4,000	4,000
*C. elegans*	16,000	16,000	4,000	4,000
*D. melanogaster*	16,000	16,000	4,000	4,000

### Architecture of 4mCPred-MTL

**FIGURE 1:**
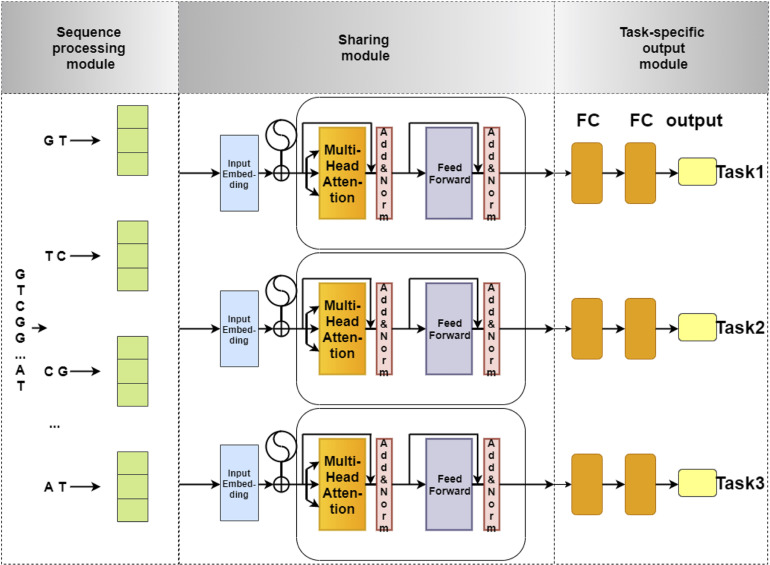
The flowchart of 4mCPred-MTL. The sequence processing module uses 2-gram to split an original DNA sequence into overlapping subsequences and converts them into feature vectors by one-hot encoding. Next, the feature vectors of subsequences are fed into the sharing module, containing a Transformer encoder and a *max-pooling* layer, to capture the sharing information among different species. Finally, the output of the sharing module is fed into the task-specific output module to predict the 4mC site of a certain species.

The network architecture of our model is illustrated in Figure 1. This network architecture consists of three main components: (i) sequence processing module, (ii) sharing module, and (iii) task-specific output module. The sequence processing module is designed to encode the DNA sequences into feature matrices by one-hot encoding (Quang and Xie, 2016; Zou et al., 2019; Dao et al., 2020a). Next, the encoded matrix is passed through a Transformer, which is a popular technique for embedding different levels of dependency relationships between subsequences. Afterward, we used a max-pooling layer to automatically measure which feature plays a key role in the target task in each unit of the Transformer. Finally, the features derived from the max-pooling layer is fed to the task-specific output module to identify 4mC sites in three species, respectively. The task-specific output module contains three parts, and each part consists of fully connected layers that are designed in terms of the size of the training set for each species. The model is implemented using Pytorch. Each module of our model is described in detail as follows.

#### Sequence Processing Module

We first employed n-gram nucleobases to define “words” in DNA sequences (Dong et al., 2006; Zeng et al., 2018; Fu et al., 2020; Lin et al., 2020; Liu X. et al., 2020; Wang et al., 2020; Yang et al., 2020; Zhang Z. Y. et al., 2021). The n-grams are the set of all possible subsequences of nucleotides. Afterward, the DNA sequences are segmented into overlapping n-gram nucleotides. The number of possibilities is 4^*n*^, since there are four types of nucleotides. To prevent the sparsity in the encoding, the n-gram number n is set to 2. For example, we split a DNA sequence into overlapping 2-gram nucleotide sequences as follows: *GTTGT…CTT*→ “*GT*,” “*TT*,” “*TG*,” “*GT*,” …, “*CT*,” “*TT*.”

For a given DNA sequence *P* with length *L*, it can be denoted as follows:

(1) $$P=R_{1},R_{2},,R_{L}$$

where *R_i_* is the *i*th word. These words are first randomly initialized and embedded by one-hot embedding, which is referred to as “word embeddings.” Here, we define the sequence of word embeddings as

(2) $${\text{x}}_{1},{\text{x}}_{2},,{\text{x}}_{L}\left(2\right)$$

where *x*_*i*_∈ℝ^*d*^ is the *d*-dimensional embedding of the *i*th word.

#### Sharing Module

##### Attention Mechanism

The attention mechanism was proposed by Bahdanau et al. (2014) in the application of neural machine translation. The Attention mechanism is somewhat similar to the idea of human translating articles, that is, paying attention to the corresponding context of our translation part. For example, we can get the hidden states of the recurrent neural network (RNN) encoder: (*h*_1_,*h*_2_,,*h*_*t*_). By assuming the current decoder hidden state is *s*_*t–1*_, we can calculate the correlation between each input position *j* and the current output position:

(3) $$\vec{c_{t}}=\left(a\,\left(s_{t-1},h_{1}\right),?\,\cdots ,a\,\left(s_{t-1},h_{T}\right)\right)$$

where *a* is a correlation operator, such as dot product. We can get the attention distribution by normalizing the $\vec{c_{t}}$. The expanding form of the attention is

(4) $${\text{a}}_{t\,j}=\frac{\text{e}\,x\,p\,\left({\text{c}}_{t\,j}\right)}{\sum_{k=1}^{T}\text{e}\,x\,p\,\left({\text{c}}_{t\,k}\right)}.$$

Therefore, attention is a weight vector. These weights represent which tokens the machine focuses on. When the attention distribution is obtained, the weight of the more important input position for the current output position is obtained, which accounts for a larger proportion when predicting the output. By introducing the attention mechanism, we can only use the final single vector result of the encoder, so that the model can focus on all the input information that is important for the next target word, and the model effect is greatly improved.

##### Transformer With Multi-Head Attention

The development of deep learning (Dao et al., 2020b; Liu Y. et al., 2020; Long et al., 2020; Naseer et al., 2020; Zhang T. et al., 2020; Zhang Y. et al., 2020) in NLP is filled with RNN and LSTM. Transformer models completely abandon the RNN and LSTM layers and only use the attention mechanism for feature extraction. After the input has been embedded to matrix form, we first use the position encoding layer. Since the model has no recurrent or convolutional layers, there is no clear relative or absolute information about the position of the word in the source sentence. In order to let the model learn the position information better, position encoding is added and superimposed on the word embedding. An encoding method using trigonometric functions maintains its position invariance.

The position encoding function can be presented as

(5) $$P\,E_{\left(p\,o\,s,2\,i\right)}=\sin\left(p\,o\,s/10,000^{2\,i/d_{m\,o\,d\,e\,l}}\right)$$

(6) $$P\,E_{\left(p\,o\,s,2\,i\,1\right)}=\cos\left(p\,o\,s/10,000^{2\,i/d_{m\,o\,d\,e\,l}}\right)$$

where *pos* is the position of each token; *2i* and *2i1* are the even-numbered and odd-numbered dimensions of each token position vector of the cardinality, respectively, where all position subscripts start from 0; and *d*_*model*_ is the dimensionality of word vector, the same as the dimensionality of encoding.

Diving into the encoder of Transformer, we will first meet the multi-head attention module. The multi-head attention is actually a combination of multiple self-attention structures. Each head learns its characteristics in different representation spaces. The first step in calculating self-attention is to construct three vectors based on the input vector of the encoder. In our task, it is the embedding of each sequence. So for each embedding, we need to create a Query matrix, a Key matrix, and a Value matrix. These three matrices are created during the training process, all from the same input. The self-attention function can be written as

(7) $$S\,A\,\left(Q,K,V\right)=s\,o\,f\,t\,m\,a\,x\,\left(\frac{Q\,K}{\sqrt{d_{k}}}\right)\,V.$$

First, we need to calculate the dot product between *Q* and *K*. To prevent the result from being too large, we will divide it by a scale of $\sqrt{d_{k}}$, which is the dimension of query and key vectors. Then a Softmax operation is implemented to normalize the result to a probability distribution, and then it is multiplied by the matrix *V* to get the weighted summation. Multi-head attention means that we can have different *Q*s, *K*s, and *V*s representations and finally combine the results. For the encoder, these basic units are concatenated, where the keys, queries, and values are all from the output of the previous layer of encoder; that is, every position of the encoder can notice all the positions of the previous layer of encoder.

After the attention is achieved, we come to the Add-and-Norm module. The “Add” in it stands for residual connection (He et al., 2016), which is designed to solve the problem of difficult training of multi-layer neural networks. By passing the information of the last layer to the next layer without difference, it can effectively focus on only the difference part. On the other hand, “Norm” is short for the layer normalization (Ba et al., 2016). It can speed up the training process and make the model converge faster by normalizing the activation value of the layer.

##### Max-Pooling Layer

The feature vector **h** of each subsequence is fed into a *max-pooling* layer to capture the most significant feature in identifying the DNA modification to represent this subsequence. Then, all the most significant features of subsequences are concatenated into a vector to represent a DNA sequence, which is shown in the following equation:

(8) $$\text{y}=m\,a\,x_{i=1}^{n}\,{\text{h}}_{i}$$

where *i* is the *i*th subsequence, *n* is the number of subsequences in a DNA sequence, and **y** is regarded as the feature vector of a target sequence. The max-pooling layer attempts to find the most important dependencies in subsequences.

#### Task-Specific Output Module

This module consists of four sets of fully connected layers corresponding to each task. In each fully connected layer with a *relu* activation function, its output is calculated by the following equation:

(9) $${\text{f}}_{i}^{j}=r\,e\,l\,u\,\left({\text{W}}_{i}^{j}\,{\text{f}}_{i-1}^{j}\,{\text{b}}_{i}^{j}\right)$$

where ${\text{f}}_{i-1}^{j}$ is the output of the previous layer of *j*th task, ${\text{f}}_{i}^{j}$ is the current layer output of *j*th task, ${\text{W}}_{i}^{j}$ is the weight matrix, and ${\text{b}}_{i}^{j}$ is the bias vector. In each layer, the “batch normalization” technique was used to improve generalization performance (Cheng and Baldi, 2006). Finally, a *softmax* layer is added on the top of final output **f**^*j*^ to perform the final prediction. Note that the parameters of different sets of the fully connected layer are designed differently in terms of the amount of data of the corresponding task.

### Training

The task-specific features, **y**, generated by the sharing module, are ultimately sent into one set of fully connected layers in terms of it belonging to which task. For classification tasks, we used binary cross-entropy loss function as the objective:

(10) $$l=\frac{1}{N}\,\sum_{i}-\left[y_{i}\,l\,o\,g\,\left(p_{i}\right)\,\left(1-y_{i}\right)\,l\,o\,g\,\left(1-p_{i}\right)\right]$$

where *N* denotes the number of training samples, *y_i_* denotes the label (i.e., 1 or 0) of sample *i*, and *p_i_* denotes the probability that sample *i* is predicted to be positive. Our global loss function is the linear combination of loss function for all tasks:

(11) $$l_{a\,l\,l}=\sum_{k=1}^{k}\alpha_{k}\,l_{k}$$

where α_*k*_ is the weight for task *k*.

### Evaluation Metrics

Here, we adopted four commonly used metrics to measure the performance of the proposed method and existing methods, including sensitivity (SN), specificity (SP), overall accuracy (ACC), and Matthew’s correlation coefficient (MCC) (Wei et al., 2014, 2017a,c, 2018c, 2019a,c,d, 2020b; Feng et al., 2019; Jin et al., 2019; Zou et al., 2019; Hong et al., 2020; Qiang et al., 2020; Su et al., 2019a,b, 2020a; Zhao et al., 2020). They are formulated as follows:

$$S\,N=\frac{T\,P}{T\,P+F\,N}$$

$$S\,P=\frac{T\,N}{T\,N+F\,P}$$

$$A\,C\,C=\frac{T\,P+T\,N}{T\,P+F\,N+T\,N+F\,P}$$

$$M\,C\,C=\frac{\left(T\,P\times T\,N\right)-\left(F\,P\times F\,N\right)}{\sqrt{\left(T\,P+F\,N\right)=\left(T\,P+F\,P\right)=\left(T\,N+F\,N\right)=\left(T\,N+F\,P\right)}}$$

where TP, TN, FP, and FN represent the numbers of true positives, true negatives, false positives, and false negatives, respectively. MCC and ACC are two metrics used to evaluate the overall prediction ability of a predictive model. In addition, we used the ROC curve to intuitively validate the overall performance. The AUC is to quantitatively evaluate the overall prediction performance of the model (Tang H. et al., 2018; Jin et al., 2020; Zeng et al., 2020; Cai et al., 2021; Zhang D. et al., 2021). The AUC ranges from 0.5 to 1. The higher the AUC score, the better the performance of the model.

## Results and Discussion

### Performance Comparison With Other Single-Task State-of-the-Art Methods

To demonstrate the effectiveness of the proposed method, we compared its performance with four other existing single-task state-of-the-art methods on the benchmark dataset, including 4mcPred-IFL (Wei et al., 2019b), 4mcPred_SVM (Wei et al., 2019a), and Deep4mcPred (Zeng and Liao, 2020). It is worth noting that among the three competing methods, except the method Deep4mcPred using deep learning technique, other methods all use traditional machine learning to train the respective models by hand-made features extracted from original DNA sequences. For a fair comparison, the source codes of these methods are used to carry out independent tests on our benchmark dataset.

The results of different methods are listed in Table 2. As shown in Table 2, we can see that for all species (i.e., *A. thaliana*, *C. elegans*, and *D. melanogaster*), our proposed method significantly outperform all other single-task competing methods in terms of SN, ACC, and MCC, with the only exception that the value of SP of our proposed method is lower than those of other methods. Specifically, for the species *A. thaliana*, when compared to the second-best method Deep4mcPred, our proposed method achieves an SN of 89.7%, an ACC of 86.5%, and an MCC of 0.728, yielding a relative improvement over Deep4mcPred of 10.33, 4.09, and 10.14%, respectively. However, Deep4mcPred does have a higher SP of 84.8, where our method only reaches an SP of 84.2. For the species *C. elegans*, compared to all competing methods, our proposed method achieves great improvement in terms of SN, ACC, and MCC, which are 6.06, 4.24, and 12.73% higher than that of the runner-up Deep4mcPred. For the species *D. melanogaster*, our proposed method also gets the best performance among all methods, achieving SN of 88.0%, ACC of 86.0%, and MCC of 0.722. Note that although the SP of our proposed methods is worse than those of other methods, the other three metrics are all higher than any competing single-task method. Therefore, we can conclude that our proposed method can achieve the best predictive performance for detecting 4mC sites in multiple species. The reason may be that in our method, we used the Transformer technique to learn more discriminative features based on multi-task learning that can leverage useful information among multiple related learning tasks to help learn a more accurate learner for each task, while the competing methods only use the information from one task. So the results are not surprising that our method achieves the best performance when using multi-task learning.

**TABLE 2:** Performance comparison of the proposed method and existing single-task 4mC predictors.

Species	Method	SN (%)	SP (%)	ACC (%)	MCC
*A. thaliana*	4mcPred-IFL	70.4	**84.9**	77.7	0.559
	4mcPred_SVM	72.3	81.1	76.7	0.536
	Deep4mcPred	81.3	84.8	83.1	0.661
	Proposed	**89.7**	83.6	**86.5**	**0.728**
*C. elegans*	4mcPred-IFL	45.4	79.4	62.4	0.263
	4mcPred_SVM	43.7	75.4	59.5	0.201
	Deep4mcPred	75.6	**88.5**	82.0	0.646
	Proposed	**83.8**	83.2	**83.3**	**0.665**
*D. melanogaster*	4mcPred-IFL	65.5	**87.6**	76.5	0.544
	4mcPred_SVM	65.8	84.5	75.1	0.511
	Deep4mcPred	84.6	84.8	84.7	0.693
	Proposed	**88.0**	84.1	**86.0**	**0.722**

*The bold denotes the best performance.*

### Effect of Multi-Task Learning

**FIGURE 2:**
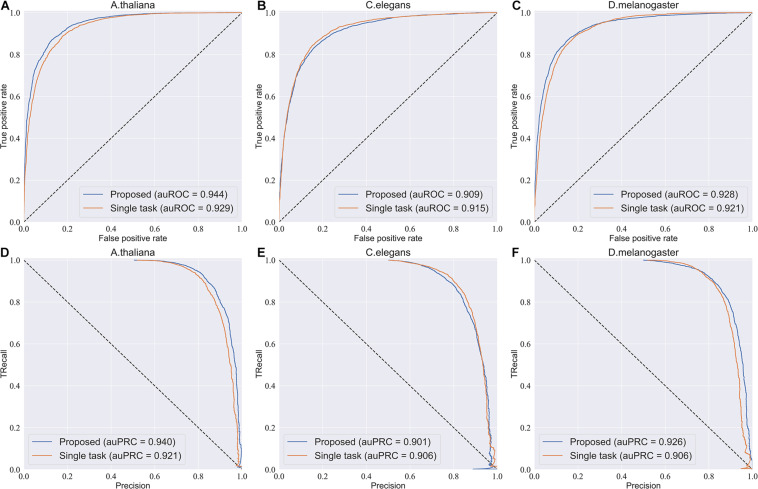
ROC curves and PR curves of the model using multi-task learning and the model not using multi-task. **(A–C)** The ROC curves of the two models in three species. **(D–F)** The PR curves of the two models in three species.

To investigate the efficiency of the multi-task learning technique, we compared the method using multi-task learning, namely, our proposed method, with the method not using multi-task learning. The comparative results obtained are shown in Table 3. From Table 3, we can see that the method using multi-task learning outperforms the method not using multi-task learning in the species *A. thaliana* and *D. melanogaster*, with only one exception in the species *C. elegans*. in which the performance of the method using multi-task learning is slightly worse than the methods not using multi-task learning. To be specific, for the species *A. thaliana*, the SN, ACC, and MCC of the method using multi-task learning are 3.46, 1.29, and 2.82% higher than those of the method not using multi-task learning, while the SP of the method not using multi-task learning is lower. For *D. melanogaster*, the method using multi-task learning improves the performance from 85.7 to 88.0% in terms of SN, 84.0–84.1% in terms of SP, 84.9–86.0% in terms of ACC, and 69.8–72.2% in terms of MCC. For a more intuitive comparison, we further compared their ROC curve s and PR (precision-recall) curves, which are illustrated in Figure 2. We can observe that except in the species *C. elegans*, the method using multi-task learning achieves the best values of auROC and auPRC in the other species. When using multi-task learning, even if the performance of our method is not good in one species, the performance is improved in the other species. Therefore, we can conclude that employing the multi-task learning technique in a feature learning scheme can improve the feature representation ability and predictive performance because the multi-task learning technique aims to enhance the performance of each task by sharing information between related tasks so that they complement each other.

**TABLE 3:** Performance comparison with the model not using the multi-task learning.

Species	Method	SN (%)	SP (%)	ACC (%)	MCC
*A. thaliana*	Single-task	86.7	**84.2**	85.4	0.708
	Proposed	**89.7**	83.6	**86.5**	**0.728**
*C. elegans*	Single-task	**85.9**	82.8	**84.4**	**0.688**
	Proposed	83.8	**83.2**	83.3	0.665
*D. melanogaster*	Single-task	85.7	84.0	84.9	0.698
	Proposed	**88.0**	**84.1**	**86.0**	**0.722**

*The bold denotes the best performance.*

### Analysis of Features Extracted From Multi-Task Learning Method on the Test Dataset

Discriminative features play a crucial role in developing a predictive tool with high accuracy. To investigate whether the features learning by our method is more discriminative, we compared them with five traditional hand-made feature descriptors, including ENAC, di-nucleotide composition (DNC), composition of k-spaced nucleic acid pairs (CKSNAP), electron–ion interaction pseudopotentials of trinucleotide (EIIP), and electron–ion interaction pseudopotentials of trinucleotide (PseEIIP). On the test dataset, all the features are evaluated with a 10-fold cross-validation technique by using three basic machine learning classifiers, including random forest (RF), SVM, and LightGBM.

**FIGURE 3:**
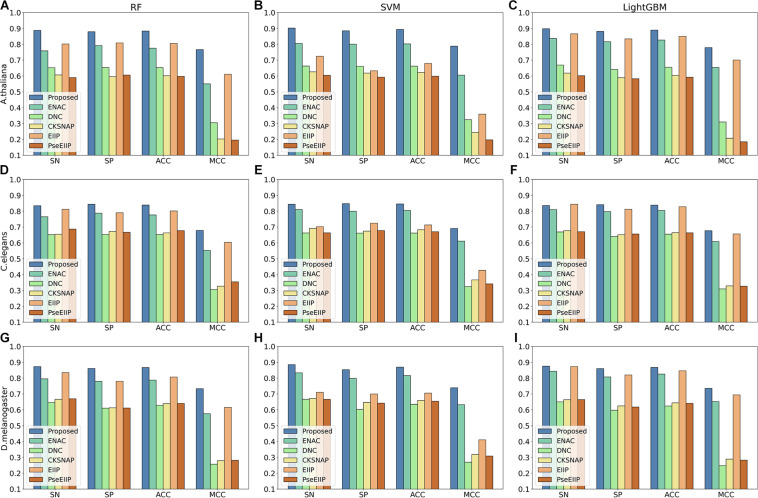
The 10-fold cross-validation results of the Proposed, ENAC, DNC, CKSNAP, EIIP, and PreEIIP methods are based on the three basic classifiers for each species. **(A–C)** The results of the species *A. thaliana*. **(D–F)** The results of the species *C. elegans*. **(G–I)** The results of the species *D. melanogaster.*

The comparison results are illustrated in Figure 3. As shown in Figure 3, we can observe that for each species, the features extracted by our proposed method achieve the best performance among other traditional hand-made features in terms of the four metrics on every basic classifier, especially on the classifiers RF and SVM, indicating that the features generated by our proposed method are more effective for 4mC sites prediction in different species and are more suitable for most of the common classifiers.

**FIGURE 4:**
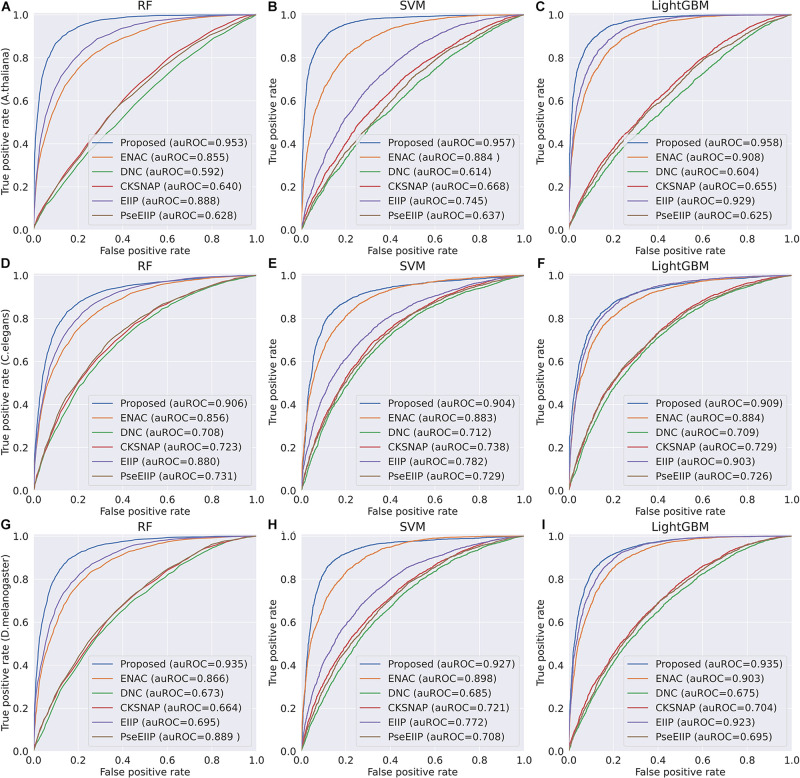
ROC curves of the Proposed, ENAC, DNC, CKSNAP, EIIP, and PreEIIP methods are based on the three basic classifiers for each species. **(A–C)** The results of the species *A. thaliana*. **(D–F)** The results of the species *C. elegans*. **(G–I)** The results of the species *D. melanogaster.*

**FIGURE 5:**
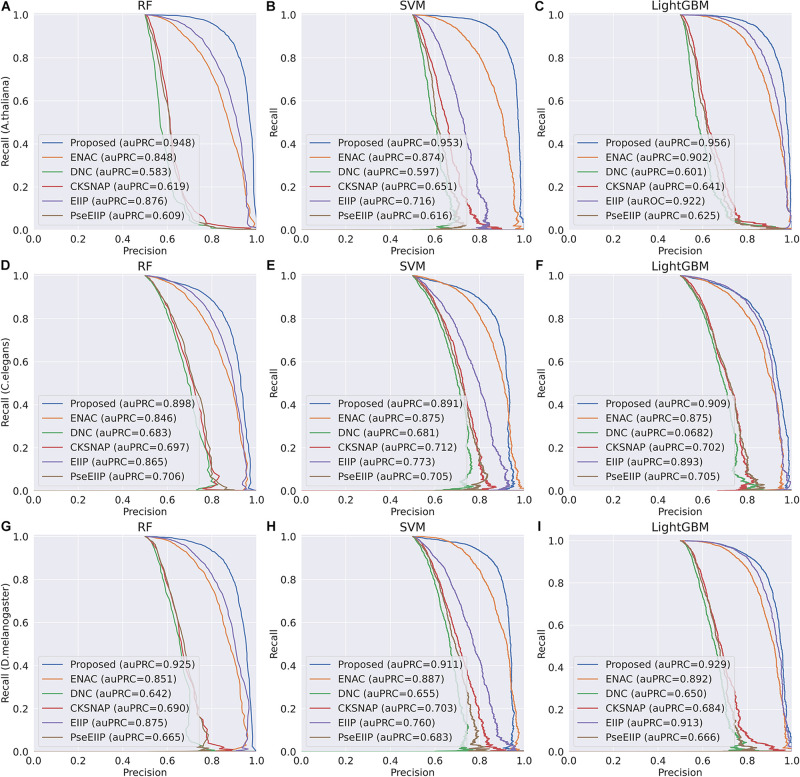
PR curves of the Proposed, ENAC, DNC, CKSNAP, EIIP, and PreEIIP methods are based on the three basic classifiers for each species. **(A–C)** The results of the species *A. thaliana*. **(D–F)** The results of the species *C. elegans*. **(G–I)** The results of the species *D. melanogaster.*

In the feature learning scheme, we used the transformer network to learn the related information between DNA subsequences and added a max-pool layer to judge which feature plays a key role in detecting 4mC sites in each subsequence. Moreover, the multi-task learning technique was exploited to capture sharing information contained in multiple tasks to help learn a more discriminative and effective feature to represent DNA sequences for 4mC sites prediction. Therefore, the proposed method significantly outperforms other traditional handcraft features, which needs prior knowledge. Figures 4, 5 illustrate the ROC and PR curves of different features. It can be also seen that our learned features are more effective than existing handcraft features, further demonstrating that our model can capture more useful information than existing feature algorithms.

## Conclusion

In this study, we have established a predictor called 4mcPred-MTL, using Transformer-based multi-task learning to predict DNA 4mC modifications in multiple species. To the best of our knowledge, this is the first 4mC predictor that can perform the prediction task for different species on a single run. Importantly, our predictor shows better performance as compared to state-of-the-art prediction tools on independent test, demonstrating the superiority of our model. In particular, via feature comparative analysis, we found that our model can sufficiently capture better characteristics of 4mC sites as compared to existing commonly used feature descriptors, demonstrating the strong feature learning ability of our model. We expect that our model can be a useful predictor for research communities of interest. In addition, we provide a new way to predict multi-species sequence prediction analysis, which can be extended to other bioinformatics fields (Ding et al., 2016a,b, 2019a,b,c,d, 2020a,b,c; Liu et al., 2017; Wei et al., 2017a,b,c, 2018a,b,c, 2020a; Jiang et al., 2018; Jin et al., 2019; Manavalan et al., 2019a,b; Su et al., 2019b, 2020b,c; Wang et al., 2019, 2021a,b; Dai et al., 2020; Guo et al., 2020a,b; Song et al., 2020; Zou et al., 2020; Yang et al., 2021).

## Data Availability Statement

Publicly available datasets were analyzed in this study. This data can be found here: http://server.malab.cn/Deep4mcPred/Download.html.

## Author Contributions

RZ surveyed the algorithms and implementations, preprocessed the datasets, and performed all the analyses. SC and ML designed the benchmarking test. All the authors have written, read, and approved the manuscript.

## Conflict of Interest

The authors declare that the research was conducted in the absence of any commercial or financial relationships that could be construed as a potential conflict of interest.
